# Primary astrocytes as a cellular depot of polystyrene nanoparticles

**DOI:** 10.1038/s41598-025-91248-w

**Published:** 2025-02-22

**Authors:** Kamil Adamiak, Marta Sidoryk-Węgrzynowicz, Beata Dąbrowska-Bouta, Grzegorz Sulkowski, Lidia Strużyńska

**Affiliations:** 1 Laboratory of Pathoneurochemistry, Department of Neurochemistry, Mossakowski Medical Research Institute, Polish Academy of Sciences, 5 Pawińskiego Str, 02-106 Warsaw, Poland; 2 Doctoral School of Translational Medicine, Centre of Postgraduate Medical Education, 99/103 Marymoncka Str., 01-813 Warsaw, Poland

**Keywords:** Polystyrene nanoparticles, Neurotoxicity, Nanotoxicity, Cell viability, Glia activation, Cellular internalization, Astrocyte phagocytosis, Cell-particle interactions, Environmental impact, Cell death in the nervous system

## Abstract

The continuous increase in plastic production has resulted in increased generation of microplastic particles (MPs), and nanoplastic particles (NPs). Recent evidence suggests that nanoplastics may be a potent neurotoxin because they are able to freely cross the blood–brain barrier and enter the brain. Therefore, the cytotoxic effects of polystyrene nanoparticles (PS-NPs) on cellular systems of cerebral origin should be thoroughly investigated. The aim of the current study is to evaluate the cytotoxic potential of 25 nm PS-NPs on in vitro cultured cells such as primary astrocytes, neurons and their co-cultures established from the cerebral cortex of Wistar pups. The results show that PS-NPs are internalized in both neurons and astrocytes, inducing time- and concentration-dependent cytotoxic effects. However, quantification of fluorescence intensity indicates cell type-dependent differences in the efficiency of PS-NPs uptake. Astrocytes are several times more efficient at accumulating PS-NPs than neurons, and this is a phagocytosis-dependent process. Moreover, the high rate of PS-NPs internalization during prolonged exposure (72 h) promotes astroglial activation, as assessed by analysis of GFAP expression and immunocytochemical imaging. The results show that astroglia act as a cellular depot of PS-NPs to protect neurons. However, once the critical threshold is exceeded, astroglia become overactivated and can lose their protective functions. These results highlight the importance of further research on the mechanisms underlying nanoplastic-induced cellular toxicity, which may have implications for understanding the broader impact of plastic pollution on neurological functions.

**Supplementary Information:**

The online version contains supplementary material available at 10.1038/s41598-025-91248-w.

## Introduction

Plastic pollution has recently become a serious environmental problem as plastic waste has been lingering for decades, contributing to the destruction of ecosystems^1–3^. Recent studies have highlighted the global impact of plastic pollution on the economy, the environment and, importantly, human health^4,5^. The continuous increase in the production of plastics, including polystyrene (PS), and the generation of plastic waste leads to the extensive formation of microparticles and nanoparticles, which are formed under the influence of mechanical forces and physical factors such as UV light^1,6^. The formation of polystyrene nanoparticles (PS-NPs) has been observed during the degradation of everyday polystyrene products, highlighting their widespread presence in the environment^7,8^. MPs and NPs entering the environment from various industrial processes, improper waste management, and disintegration of plastic items^7–9^, constitute direct sources of oral, dermal and inhalation exposure. These plastic particles have also been shown to be ingested by marine species, indicating their potential to adversely affect human health via the food chain^4,10,11^.

Particles with a diameter less than 100 nm have been reported to pose health risks due to their unique set of characteristics, such as high reactivity, potential interactions with biological systems, and toxicity. These physicochemical properties of NPs can induce adverse effects in biological systems by interacting with proteins, DNA, lipids, membranes, organelles, and biological fluids^12–14^. Their small size allows them to penetrate cell membranes, enabling them to enter the bloodstream and reach various organs^14,15^.

Plastic waste is primarily composed of synthetic organic polymers, including polystyrene, a widely produced and utilized polymer in everyday items^8^. In vitro studies using cultured human cell lines have demonstrated the potential of PS-NPs to penetrate biological membranes^4,16^ and interact with cellular structures^1,17^. Studies using animal models have shown that PS-NPs are able to cross the placenta and are absorbed by fetal organs. This induces apoptosis, inflammation, and structural disorders in various tissues^1,18,19^. Moreover, the ability of PS-NPs to overcome the blood–brain barrier (BBB) raises questions about the toxic potential of PS-NPs in the human nervous system^4,20^, which remains largely unknown.

As the world struggles with the growing problem of plastic pollution, understanding the interrelationships between PS-NPs and neurological health is essential for health organizations to make informed decisions and implement environmentally-friendly practices. Therefore, the main objective of the current research is to evaluate the effect of PS-NPs on primary astrocytes, neurons and co-cultures of these cells, and to identify potential cell type-specific cytotoxic effects of PS-NPs. To investigate the potential toxic effects of nanoplastics in brain-derived cells, PS-NPs spheres with a diameter of 25 nm were added into the appropriate cell culture media at concentrations ranging from 0.5 to 50 µg/mL. Since astrocytes are key regulators of CNS functions, exerting either neuroprotective or neurotoxic functions, we focus on the astroglial response under conditions of PS-NPs exposure.

We chose small-sized NPs (25 nm) because the impact of nanoplastics on organisms and environmental processes is less known than that of microplastics. The characteristics of small particles allow nanoplastics to bypass the traditional processes of wastewater treatment and enter water systems, likely increasing the potential for human exposure. They also tend to cross physiological barriers more easily than larger plastic particles, which can also increase the likelihood of exposure and toxic effects^21^. Moreover, amino-functionalized particles (PS–NPs-NH_2_) are more toxic because they can bind to the lipid bilayer on the cell membrane with high affinity, promoting cellular uptake via endocytosis. The ability of this type of NPs to exist in the environment for a long time significantly increases their potential bioavailability and cell penetration^22^.

The amount of plastic particles to which humans are exposed in the environment is a widely discussed topic, especially since they are difficult to quantify in environmental matrices. The inability to accurately detect micro- and nanoplastics with current methods makes estimating environmental concentrations challenging^22,23^. Based on the literature, the average measurable concentration of plastic particles in human blood was 1.6 µg/mL^24^. Therefore, we used PS-NPs concentration of 1 µg/mL in our study. Considering the ability of NPs to accumulate in cells over time, we additionally used higher concentrations (25 and 50 µg/mL), which allows determining the toxic effect of increasing concentrations of PS-NPs.

## Materials and methods

### Characteristics of PS-NPs

Amino-functionalized polystyrene nanoparticles (25 nm PS-NPs; Cat. No. PST25A) and fluorescently functionalized PS-NPs (Cat. No. FGP25A) with a green fluorescence spectrum and excitation/emission wavelength of 460/500 nm used in this study were purchased from Lab261 (Palo Alto, CA, USA) in a 10 mg/mL PS-NPs stock solution. The size distribution and spherical shape of the nanoparticles were confirmed using transmission electron microscopy JEM-1011 (JEOL, Japan) equipped with an EDS INCA (Oxford) model analyzer. The studies were performed in the Electron Microscopy Research Unit of Mossakowski Medical Research Institute, Polish Academy of Sciences (MMRI PAS) (Warsaw, Poland).

The hydrodynamic diameter and zeta potential of particles in aqueous dispersions were determined using a Malvern Zetasizer instrument (Nano ZS, UK) equipped with a 4-mW He–Ne laser (λ = 632.8 nm) as the light source. The scattering angle was set at 173°, and the solutions were allowed to equilibrate at selected temperatures for 5 min prior to measurement. To assess zeta potential, a folded capillary cell with gold electrodes was utilized. The analyzer computed the zeta potential based on the electrophoretic mobility, employing the Henry equation and the Smoluchowski approximation. pH levels were monitored using a pH/ion meter (Mettler Toledo, model SevenGo-SG2). The final pH value was recorded immediately before the measurements, and the ionic strength was maintained at a constant level (0.01 M NaCl). Analyses were performed in collaboration with the Biological and Chemical Sciences Center, University of Warsaw, Warsaw, Poland.

### Cell cultures

All cell culture materials were obtained from Thermo Fisher Scientific (Waltham, MA, USA). Primary astrocytes and neurons were isolated from the cerebral cortex of newborn Wistar rats on the first postnatal day. Briefly, decapitation of rats was performed, followed by isolation of the cortex and triturating it in Hanks Balanced Salt Solution (HBSS; Cat. No. 14060040). Cells were further dissociated by incubating the cell suspension in Trypsin–EDTA 0.05% (Cat. No. 25300096) in HBSS at 37˚C for 30 min, and fetal bovine serum (FBS; Cat. No. 10500064) was added before centrifugation at 230 × g (Eppendorf Centrifuge 5804 R). To obtain primary astrocytes, the pelleted cells were re-suspended in Dulbecco’s Modified Eagle’s Medium with Earle’s salts (DMEM; Cat. No. A4192101), supplemented with 5% FBS, penicillin and streptomycin (Cat. No. 15070063), and plated in non-coated flasks for two days. Then the astrocytes were supplemented with DMEM medium enriched with 1% G5 supplement (Cat. No. 17503012) and allowed to grow for one week to reach confluence. A confluent monolayer of astrocytes was re-plated onto 96-well plates at a density of 1 × 10^4^ cells per well, and 24-well plates at a density of 5 × 10^4^ cells per well. The cells obtained using this protocol were identified as positive for glial fibrillary acidic protein (GFAP), a marker of astrocytes.

Primary neurons were plated in poly-D-lysine (Cat. No. A3890401) coated 96- and 24-well plates, at a density of 5 × 10^4^ and 3 × 10^5^ cells per well, respectively. In contrast, neurons were cultured in Neurobasal medium (Cat. No. 21103049) supplemented with 2% MACS NeuroBrew-21 (NB-21; Miltenyi Biotech; Cat. No. 130-097-263) for the first five days and on the second day after plating, 2.5 μM cytosine arabinoside (AraC; Merck; Cat. No. C1768) was added to the cultures to impede non-neuronal cell growth. Subsequently, the medium was changed to Neurobasal medium supplemented with 2% B-27 supplement (Cat. No. 17504044) for the next five days. The cells obtained using this protocol were verified as being positive for expression of microtubule-associated protein 2 (MAP2). To obtain co-cultures, astrocytes were passaged around day 10 with neurons cultured according to the above-mentioned protocol. The proportion of the cells was about 2:1 (astrocytes:neurons). All cultures were maintained at 37˚C under an atmosphere containing 5% CO_2_.

### Immunocytochemistry and microscopic visualization

To monitor the cellular uptake of PS-NPs, the cells were first incubated with different concentrations of PS-NPs labelled with a green fluorophore for 24, 48, and 72 h. For immunocytochemistry, the cells were first washed with PBS and then fixed in 4% paraformaldehyde for 20 min to preserve their cellular structures. To enable cell penetration of antibodies, the cells were permeabilized using 0.1% Triton X-100 for 30 min. Non-specific binding was blocked by incubating the cells with 5% bovine serum albumin (Merck; Cat. No. A7030) for 1 h. Primary astrocytes were then incubated overnight with the primary antibody—anti-glial fibrillary acidic protein (GFAP; 1:500; Merck; Cat. No. G3893) in PBS-T containing BSA. Primary neurons were incubated with anti-microtubule-associated protein-2 (MAP-2; 1:500; Merck; Cat. No. M3696) antibody, respectively. After overnight incubation, the cells were then incubated with a fluorophore-tagged secondary antibody—Alexa Fluor 546 (1:500; Thermo Fisher Cat. No. A11030 for astrocytes, and A11035 for neurons) for 60 min in PBS-T with BSA. Additionally, primary astrocytes were labelled with secondary Alexa Fluor 488-conjugated antibody (1:750; Thermo Fisher; Cat. No. A11001). Finally, Hoechst dye (Merck; Cat. No. B2883) was added to stain the nuclei. The co-cultures were stained under similar conditions. For co-culture staining, primary astrocytes were labelled with Alexa Fluor 594-conjugated secondary antibody (Cat. No. A11005; ThermoFisher) after coating with anti-GFAP antibody (Cat. No. G3893; Sigma), while primary neurons were labelled with anti-MAP-2 antibody (Cat. No. M3696; Sigma) followed by Alexa Fluor 546 secondary antibody (Cat. No. A11035; ThermoFisher).

The internalization of PS-NPs in primary cells was visualized using an Axio Vert.A1 fluorescence microscope and LSM 780/ELYRA PS.1 confocal system (Carl Zeiss, Jena, Germany). Microscopic analysis was performed in cooperation with the Environmental Laboratory of Laser Microscopic Techniques (LLTM) at MMRI PAS. Quantification of fluorescence intensity was done with approximately 20 microscopy images (objective 40x) for each set of experimental conditions, using the mean fluorescence intensity of PS-NPs or GFAP, calculated by Zen Software (Carl Zeiss, Jena, Germany).

Confocal images of control astrocytes and astrocytes exposed to 50 µg/mL PS-NPs for 72 h were stored as TIFF files and converted to 8-bit images to quantify astrocyte morphology. Sholl analysis^25^ was conducted using the Fiji ImageJ with plugin SNT (https://imagej.net/plugins/snt/) based on the website (https://imagej.net/plugins/sholl-analysis). The geometric center (cell soma) was manually set and marked using the line tool in ImageJ. The analysis quantified the branching complexity. The mean value of the intersections and Sholl decay were chosen as the parameters estimating the density of astrocyte processes.

### Phagocytic activity of primary astrocytes

To determine whether internalization of PS-NPs in primary astrocytes depends on phagocytosis, we used cytochalasin-D (Cyt. D; Cat. No. C8273; Sigma) to inhibit this process via actin polymerization. Cells were pretreated with 2 μM of inhibitor for 30 min prior to the addition of 50 μg/mL PS-NPs for 24 h. After this time, cells were subjected to immunocytochemical procedure and visualization of internalized PS-NPs as described above. The intensity of PS-NP-derived green fluorescence was measured in approx. 15 microscopy images and calculated by Zen Software (Carl Zeiss, Jena, Germany).

### Cell viability

The primary astrocytes and neurons were exposed to different concentrations (0.5; 1; 25; 50 µg/mL) of PS-NPs dispersed in appropriate cell culture medium for 6, 24, 48 and 72 h. To obtain working concentrations of PS-NPs, the stock solution (10 mg/mL) was diluted with DMEM (for astrocytes) and neurobasal medium (for neurons) and vortexed prior to use. For cytotoxicity assays, the cells were plated in 96-well plates and cultured in 100 μL of complete growth medium. The control cells were maintained in the same volume of appropriate medium without the addition of PS-NPs. The toxicity of PS-NPs to primary cells was evaluated using MTT and LDH assay kits performed according to the manufacturer’s instructions described below.

#### MTT cytotoxicity assay

The Invitrogen™ CyQUANT™ MTT Cell Proliferation Assay Kit (Cat. No. M6494) was used to determine cell viability based on the conversion of water-soluble 3-(4,5)-dimethylthiazol-2-yl)-2,5-diphenyltetrazolium bromide (MTT) to insoluble formazan by the redox potential in viable cells^26^. After exposure to PS-NPs and before the assay, the medium was replaced with medium DMEM-F12 without phenol red (Thermo Fisher; Cat. No. 21041025) and 12 mM MTT was added to each well, and incubated for 2 h at 37°C. After incubation, the medium was removed and DMSO was added. The medium was then incubated further for 10 min at 37°C to solubilize the formazan. Finally, the absorbance was measured at 540 nm and relative absorbance was calculated from the absorbance of a control sample.

#### LDH cytotoxicity assay

The CyQUANT™ LDH Cytotoxicity Assay Kit by Invitrogen (Cat. No. C20301) was used to determine the cytotoxic effect of PS-NPs in cultures. Lactate dehydrogenase (LDH), a reliable indicator of cytotoxicity, is an enzyme found in various cell types. When the plasma membrane is damaged, LDH is released into the surrounding cell culture media and can be measured using a coupled enzymatic reaction. To perform the assay, sterile water was added to the set of wells with spontaneous LDH activity control. Lysis buffer was added to maximum LDH activity controls. The plate was then incubated for 45 min at 37°C in 5% CO_2_. Next, each sample medium was transferred to a new 96-well plate, and the reaction mixture was applied to each well. The plate was then incubated at room temperature for 30 min, and a stop solution was added to each well. The absorbance was measured at 490 nm and 680 nm. The LDH activity was calculated by subtracting the 680-nm absorbance (background) value from the 490-nm absorbance. Then, cytotoxicity was calculated using the following formula:

$$Cytotoxicity\; \left[ \% \right] = \frac{{\left( {PS - NPs \;treated \;LDH \;activity} \right) - \left( {Spontaneous\; LDH\; activity} \right)}}{{\left( {Maximum\; LDH \;activity} \right) - \left( {Spontaneous\; LDH \;activity} \right)}} \times 100$$

### Western blot analysis

To analyse the relative concentration of GFAP protein in primary astrocytes, cells were mixed with RIPA buffer (Thermo Fisher; Cat. No. 89900) with protease (Merck; Cat. No. P8340) and phosphatase (Merck; Cat. No. P0044) inhibitors. The mixture was boiled and centrifuged, and 15 μg of protein was loaded onto a 10% sodium dodecyl sulfate–polyacrylamide gel. Proteins were separated and transferred to nitrocellulose membranes. The membranes were incubated in a buffer containing Tris–HCl, NaCl, and Tween-20 with 5% bovine albumin and then with primary antibody against GFAP (1:800; Merck; Cat. No. G3893). Subsequently, the membranes were washed with buffer and exposed to an anti-mouse secondary antibody (1:5000; Merck; Cat. No. A2304). Beta-actin (Abcam; Cat. No. ab49900) was used to confirm the equivalent loading of each sample. Finally, the chemiluminescence signal was detected using an ECL kit (Santa Cruz; Cat. No. sc-2048) and analyzed using ImageJ software.

### Statistical analysis

All experiments were performed at least in triplicate. The data are expressed as means ± SD and a *p*-value was considered < 0.05. Inter-group comparisons were performed using the Kruskal–Wallis test with Dunn’s post hoc test, one-way and two-way ANOVA (with Tukey’s and Dunnett’s post hoc) or unpaired T-test and Mann–Whitney, as stated in the respective figure legends. GraphPad Prism (GraphPad Software, Inc., La Jolla, CA, USA) was used to prepare plots of the data.

## Results

### Characterization of PS-NPs

Polystyrene nanoparticles (PS-NPs) 25 nm in diameter, functionalized with an amine group (–NH_2_) are the subject of the present research. For imaging studies, nanoparticles with the same properties, conjugated with a green fluorochrome, were also acquired. Since the cytotoxicity of nanomaterials depends on various parameters, a series of analyses were conducted prior to the study to characterize the physicochemical properties of the commercially obtained PS-NPs.

**Fig. 1:**
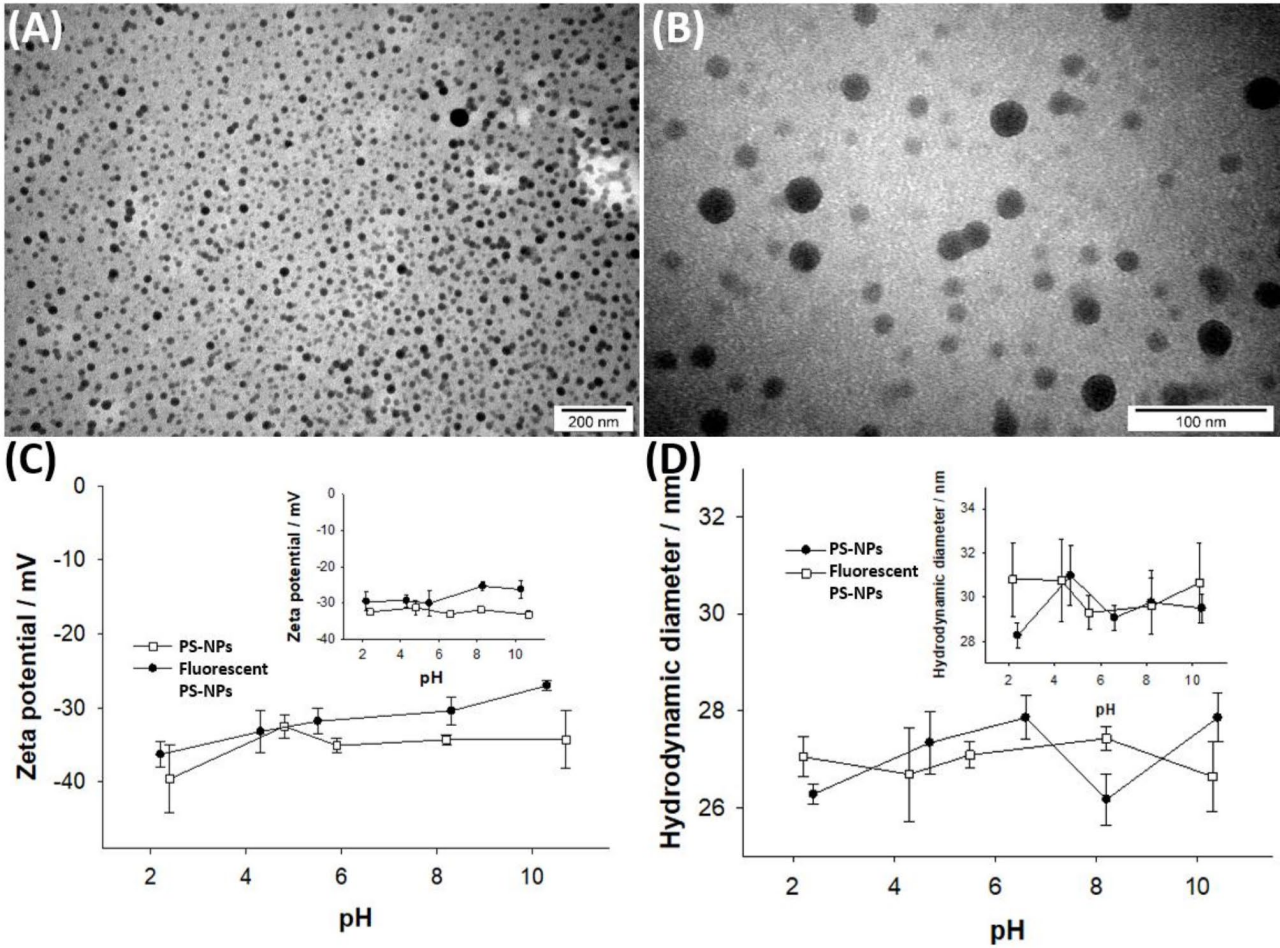
TEM images showing spherical structure and dispersity of PS-NPs used throughout the study. The scale bar is 200 nm (**A**) and 100 nm (**B**). Graphs showing zeta potential (**C**) and hydrodynamic diameter (**D**) plotted vs. pH gradient at 25 °C and 37 °C (insets), measured for unlabelled and fluorescently labelled PS-NPs. Data are means ± SD from 3 independent measurements. Ionic strength: 10 mM.

Ultrastructural analysis of the PS-NPs was carried out using transmission electron microscopy (TEM) to determine their shapes. The images confirmed that PS-NPs are mostly spherical in shape (Fig. 1A, B) and do not agglomerate. This is consistent with the information provided by the manufacturer.

The analysis of changes in zeta potential (Fig. 1C) of both unlabelled and labelled with a fluorochrome PS-NPs was performed in a pH gradient and at temperatures of 25°C and 37°C. The charge of both types of PS-NPs was negative at all pH values and temperatures tested. This indicates the stability of these particles in solution and the lack of agglomeration. The highest zeta potential, around − 25 mV, was observed for labelled nanoparticles at pH 8.3 and 37 °C, while the lowest, approximately − 40 mV, was recorded for unlabelled PS-NPs at pH 2.4 and 25 °C. In the next step, the hydrodynamic diameter (Fig. 1D) of PS-NPs at different pH values and temperatures as well as the particle size distribution under close to physiological conditions (37 °C and pH 7.4) were determined. Changes in nanoparticle size were found to be correlated with alterations in zeta potential but independent of pH. The size of both types of nanoparticles ranged from 26 to 28 nm at 25 °C and 28 to 31 nm at 37 °C. The size of PS-NPs was also measured in phosphate buffer at pH 7.4 and 37°C. The average size of unlabelled and labelled PS-NPs was approximately 37.28 ± 10.73 nm and 28.67 ± 8.47 nm, respectively. Similar results were obtained in cell culture medium.

The hydrodynamic size of colloidal particles can be calculated from the diffusivity using a mathematical equation. Particle sizes calculated in this way are generally larger than those measured by TEM^27^. Indeed, our previous size measurements by TEM revealed that the size distribution follows a Gaussian style and the average diameter of PS-NPs in the delivered batch is approximately 24 nm^28^.

### Internalization of fluorescent PS-NPs in exposed primary cell cultures

**Fig. 2:**
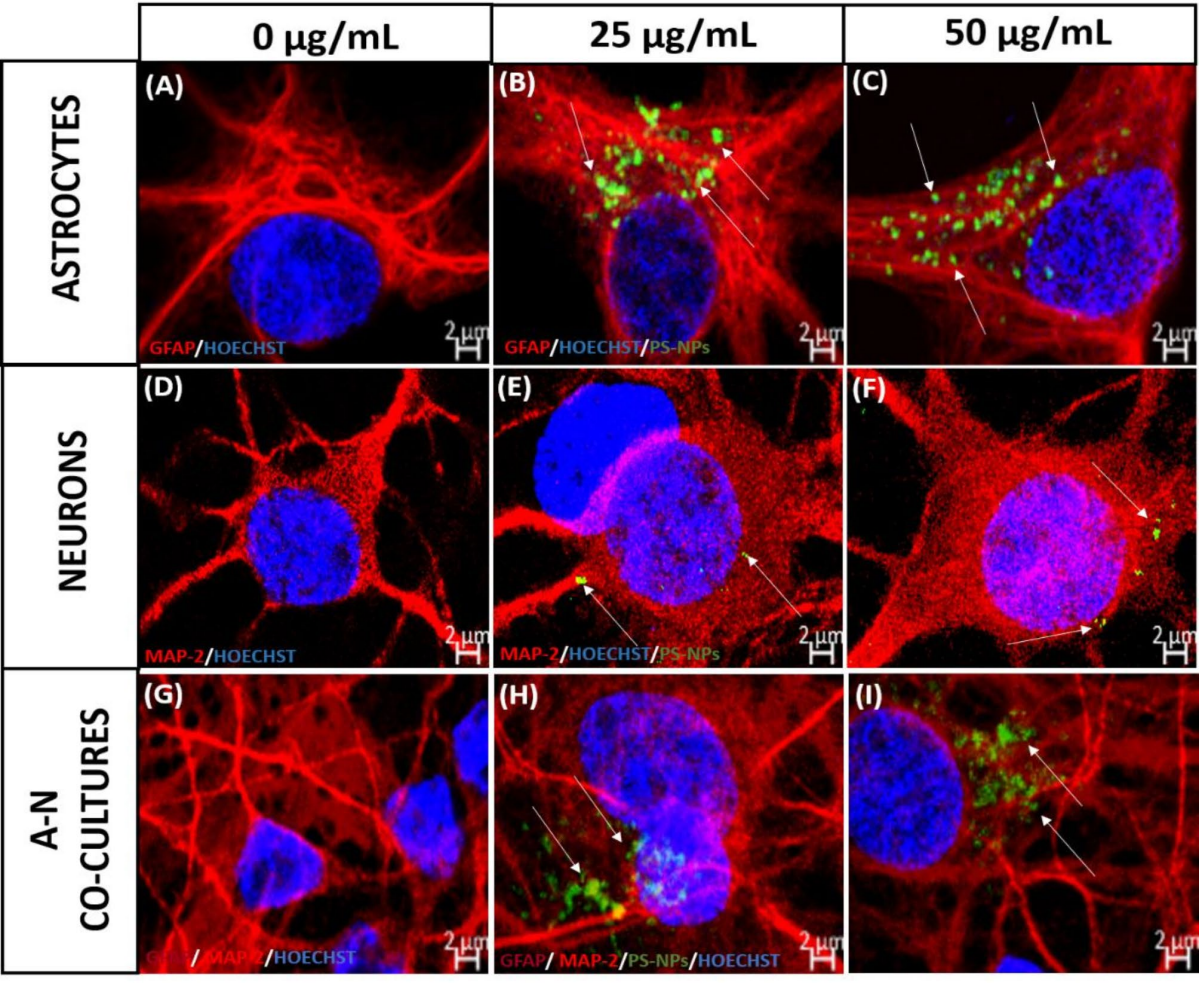
Representative confocal microscopy images showing the internalization of PS-NPs in primary astrocytes (**A**–**C**), neurons (**D**–**F**) labelled with GFAP and MAP-2, respectively, and in co-cultures of these cells (**G**–**I**). Cells treated with green fluorescent PS-NPs at concentrations of 0 µg/mL (control; **A**, **D**, **G**), 25 µg/mL (**B**, **E**, **H**) and 50 µg/mL (**C**, **F**, **I**) for 72 h. The scale bar is 2 µm. Green dots indicate accumulated particles (arrows).

We further compared the extent to which PS-NPs accumulated in exposed cells as a function of time and concentration. Primary astrocyte/neuron cultures were exposed to different concentrations of green fluorescent PS-NPs at several time points. After exposure, cells were fixed, immunocytochemically stained and observed under a confocal microscope (LSM 780 System) and fluorescence microscope (Axio Vert.A1). Fluorescence originating from PS-NPs was not observed in control astrocytes (Fig. 2A), but green dots showing a perinuclear distribution pattern were clearly visible in exposed cells, even after the first 24 h of exposure. Extensive accumulation was observed over time (Fig. 2B, C). As expected, control neurons also did not exhibit green fluorescence (Fig. 2D), whereas exposed cells displayed a different pattern of PS-NPs distribution, which accumulated randomly in different parts of the cells, including cellular processes. In contrast to astrocytes, particles were barely visible after short exposure (24 h) and at low concentrations, and were also less numerous and did not form large clusters (Fig. 2E, F). In the co-cultures the pattern of PS-NPs distribution was similar to that in astrocyte cultures (Fig. 2H, I).

**Fig. 3:**
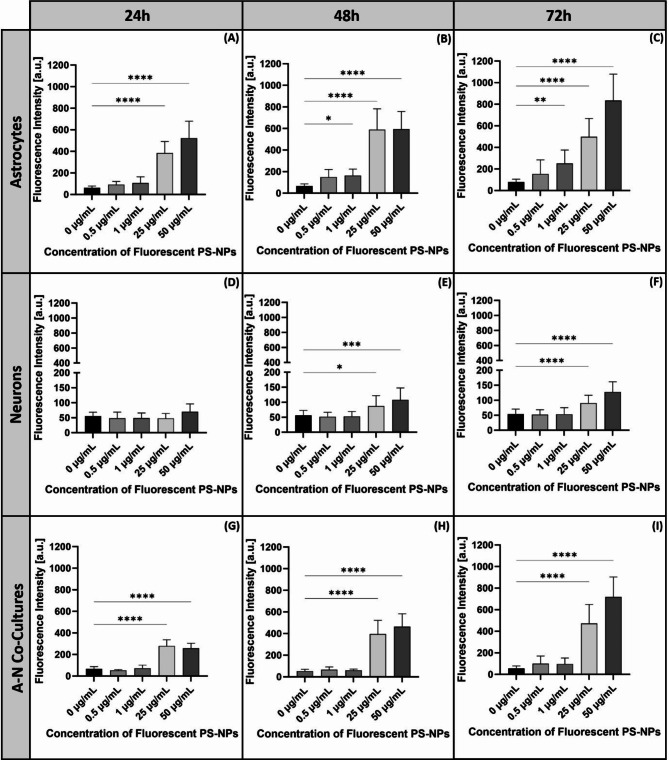
The fluorescence intensity derived from PS-NPs internalized in primary astrocytes (**A**–**C**), neurons (**D**–**F**) and co-cultures of these cells (**G**–**I**) after 24 h (**A**, **D**, **G**), 48 h (**B**, **E**, **H**) and 72 h (**C**, **F**, **I**). The studies were conducted in four biological replicates (total number of measurements in each group = 30); *p < 0.05; **p < 0.01; ***p < 0.001; ****p < 0.0001 (Kruskal Wallis test followed by Dunn’s post hoc test).

**Fig. 4:**
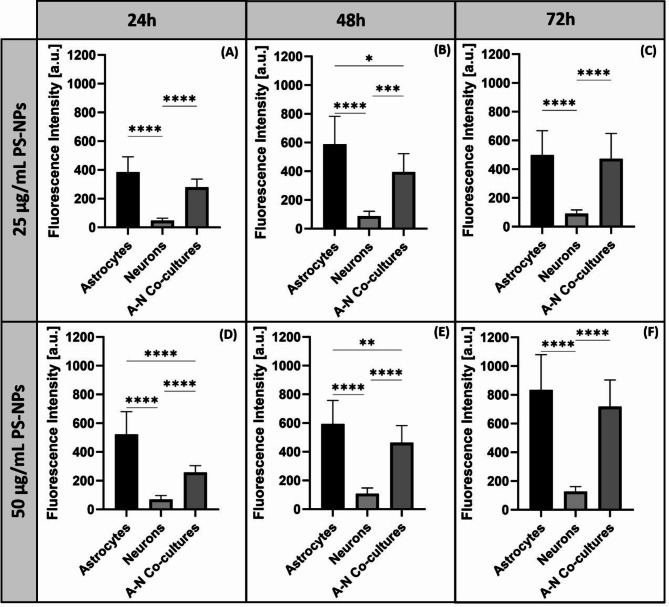
Cell type-dependent fluorescence intensity derived from PS-NPs measured in primary astrocytes, neurons and their co-cultures exposed to 25 µg/mL (**A**–**C**) and 50 µg/mL (**D**–**F**) of PS-NPs for 24, 48 and 72 h. The results are means ± SD of four independent experiments done using distinct cultures (total number of measurements in each group = 30); *p < 0.05; **p < 0.01; ***p < 0.001; ****p < 0.0001 (**A**–**B**: Kruskal Wallis test followed by Dunn’s post hoc; **C**–**F**: one-way ANOVA with Tukey’s post hoc).

The microscopic observations were confirmed by measurements of the green fluorescence intensity originating from PS-NPs. Statistical analysis revealed that entry of PS-NPs into cells depends on their concentration gradient (Fig. 3) and the type of exposed cells (Fig. 4).

The internalization of PS-NPs in astrocytes increased with increasing concentrations and time (Fig. 3A-C) and the mean fluorescence was found to be significantly different from the control value in the concentration range of 25–50 µg/mL starting from 24 h of exposure (Fig. 3A) and in the concentration range of 1–50 µg/mL after 48 and 72 h of exposure (Fig. 3B, C). The accumulation of PS-NPs in neurons was also found to be concentration-dependent (Fig. 3D–F) but significantly different from the control only after longer exposure time (48–72 h) at 25 µg/mL and 50 µg/mL. Corresponding observations were made in co-cultures of astrocytes and neurons, where statistically significant increases in fluorescence intensity compared to the control were visible starting from 24 h of exposure, but only at higher concentrations of 25 µg/mL and 50 µg/mL (Fig. 3G–I).

An important observation was the dependence of the internalization rate on the cell type. To better visualize this phenomenon, we plotted fluorescence intensity against cell type (Fig. 4). The intensity of PS-NP-derived green fluorescence measured in astrocytes exceeds that measured in neurons by several orders of magnitude (4–6 times), which means that PS-NPs are more easily internalized in astrocytes and accumulate therein in higher abundance. This effect was noted at all exposure time points and PS-NPs concentrations of 25 µg/mL (Fig. 4A–C) and 50 µg/mL (Fig. 4D–F).

It is also worth noting that the cells in mixed cultures accumulated less PS-NPs than would be expected based on the fluorescence levels recorded for the astroglial monoculture, but more PS-NPs than observed in the neuronal monocultures. This result is consistent with the analysis of the cell type ratio in the co-culture, which was 2:1 (astrocytes vs. neurons).

However, it should be noted that the fluorescence signal detected in the mixed culture is the average of all cells. Since astrocytes and neurons are often closely associated with each other in co-culture system, it is difficult to distinguish PS-NPs fluorescence signals originating from each cell type.

**Fig. 5:**
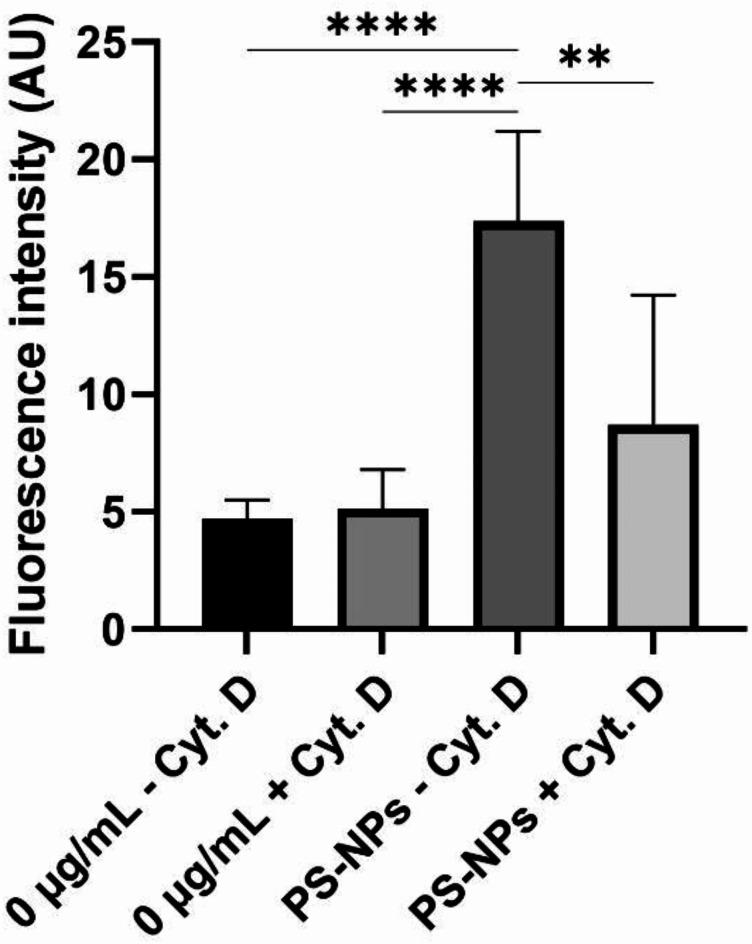
Fluorescence intensity originating from PS-NPs internalized in primary astrocytes—control (0 µg/mL) or exposed to 50 µg/mL PS-NPs for 24 h, and pre-treated (+ Cyt. D) or not (− Cyt. D) with cytochalasin D, an inhibitor of phagocytosis. The results are means ± SD of approx. 15 measurements done in each experimental group. **p < 0.01; ****p < 0.0001 (Kruskal Wallis test followed by Dunn’s post hoc test).

We next searched for the mechanism responsible for the high ability of primary astrocytes to internalize PS-NPs. To assess whether phagocytosis is involved in this process, we pretreated cells with cytochalasin D, an inhibitor of actin polymerization, 30 min before exposure to PS-NPs. Measurements of the fluorescence intensity derived from PS-NPs show that the internalization of PS-NPs in astrocytes was significantly, but not completely, suppressed by the addition of cytochalasin D compared to astrocytes not pre-treated with this inhibitor (Fig. 5). This clearly indicates that actin-dependent process underlies this internalization and phagocytosis may be one of the mechanisms involved in the uptake of PS-NPs by primary astrocytes.

### Cytotoxic effect of PS-NPs in primary astrocytes and neurons

The next step of the study was to check whether internalized PS-NPs exert adverse effects on cells. Cytotoxicity following administration of PS-NPs (in the concentration range of 0.5–50 µg/mL and time intervals of 6–72h) was assessed using the MTT and LDH assays. A short exposure time (6 h) and the lowest concentration of PS-NPs used (i.e. 0.5 µg/mL) did not affect the viability of cells cultured separately or in co-culture, regardless of the assay used.

**Fig. 6:**
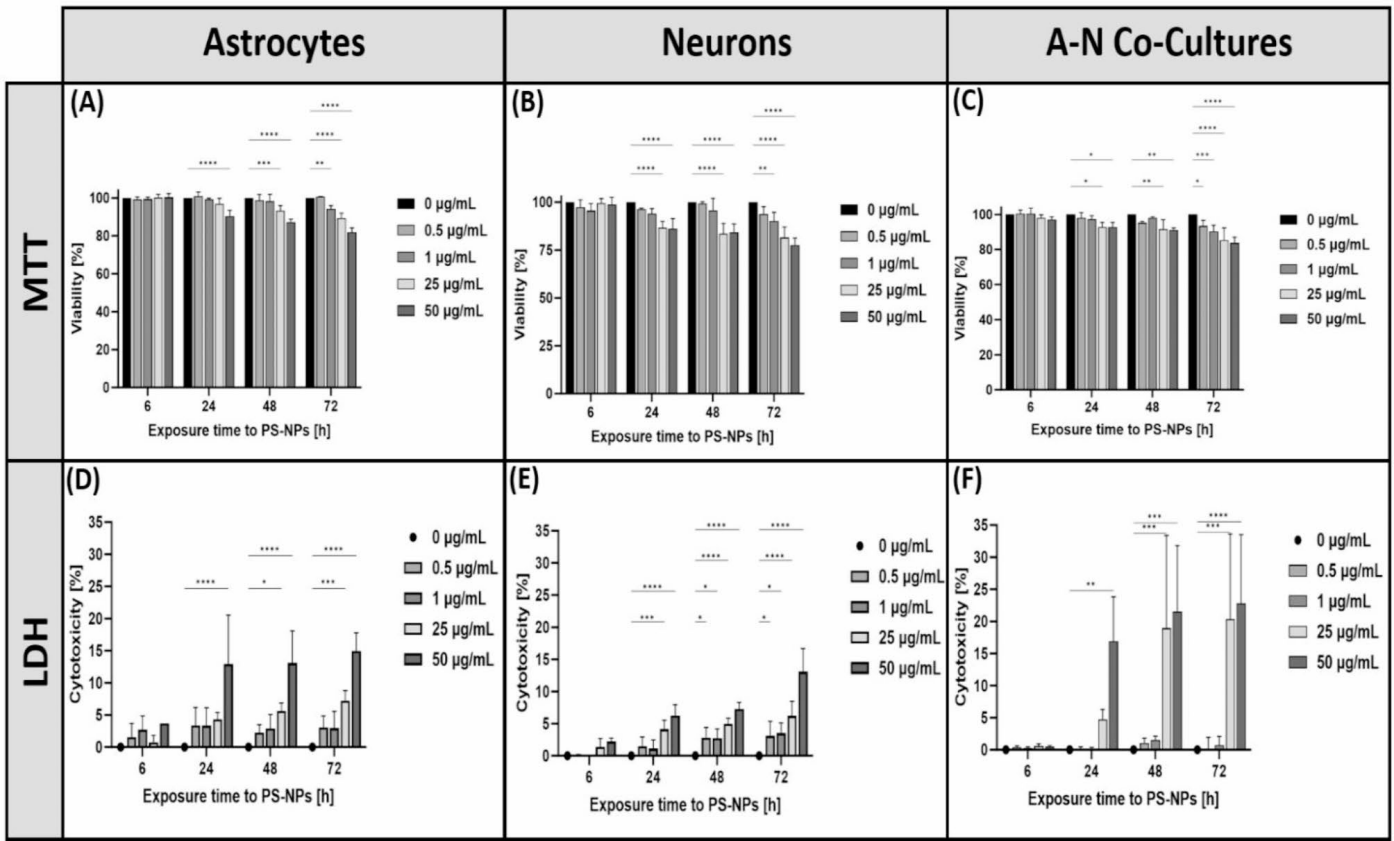
Time- and concentration-dependent cytotoxic effect of PS-NPs measured by MTT and LDH tests in primary astrocytes (**A**, **D**), neurons (**B**, **E**), and co-cultures (**C**, **F**) of these cells. The studies were conducted in at least three biological replicates (total number of measurements in each group = 15 (MTT), 30 (LDH); *p < 0.05; **p < 0.01; ***p < 0.001; ****p < 0.0001 (two-way ANOVA test followed by Dunnett’s post hoc test).

After the first 24 h of exposure, the results of the MTT test showed only a reduction in viability of astrocytes (90% of the control value; *p* < 0.0001) at a concentration of 50 µg/mL. Following prolonged exposure to PS-NPs at higher concentrations of 50 µg/mL and 25 µg/mL, astrocyte viability decreased to 87% (*p* < 0.0001) and 93% of the control value (*p* < 0.001), respectively. The lowest cell viability, 82% of the control value (*p* < 0.0001), was observed at a concentration of 50 µg/mL PS-NPs after 72 h of exposure (Fig. 6A).

For each cell type, the level of damage was found to be similar whether determined by LDH release or inhibition of MTT reduction. The highest cytotoxicity of PS-NPs towards astrocytes was observed after 72 h of exposure, at concentrations of 25 µg/mL and 50 µg/mL (Fig. 6D), where the levels of released LDH were 7% and 15% higher compared to the control, respectively (*p* < 0.0001). After 48 and 24 h of exposure to 50 µg/mL of PS-NPs, similar levels of toxicity were observed resulting in a 13% increase of LDH compared to control (*p* < 0.0001). The lower concentration (25 µg/mL) was found to induce a 6% increase in LDH after 48 h of exposure (*p* < 0.5). In summary, the cytotoxicity of PS-NPs assessed by both the MTT and LDH tests shows time- and concentration-dependent profiles, increasing over time with increasing concentration of PS-NPs.

Regarding the cytotoxic effect of PS-NPs on primary neurons, a significant decrease in viability was observed after 24 h of exposure at PS-NPs concentrations of 50 µg/mL and 25 µg/mL, reaching 86% and 87% of control cell viability, respectively (both *p* < 0.0001). After 48 h of exposure, cell viability was found to be further reduced at both concentrations of PS-NPs (*p* < 0.0001). With respect to astrocytes, the lowest viability (78%; *p* < 0.0001 vs. control) was observed after 72 h of exposure to PS-NPs at a concentration of 50 µg/mL (Fig. 6B). However, even at lower concentrations, i.e., 25 µg/mL (82%; *p* < 0.0001) and 1 µg/mL (90%; *p* < 0.01), a significant decrease in neuron viability was observed at this time point.

The maximal LDH releases in primary neurons were observed at the highest tested concentration of 50 µg/mL after 72 h of exposure (Fig. 6E), where the degree of neuron damage was 13% (*p* < 0.0001) compared to control, correlating with the MTT viability results. Moreover, all other tested concentrations of PS-NPs (i.e. 0.5, 1, 25, 50 µg/mL) were found to exhibit statistically significant toxicity after 72 h. Similar results were obtained after 48 h of exposure. After short-term exposure (24 h), toxic effects were observed only at concentrations of 50 µg/mL (6%; *p* < 0.0001) and 25 µg/mL (4%; *p* < 0.001). The results correlate with those obtained in astrocyte cultures, indicating that cytotoxicity of PS-NPs to neurons increases in a dose- and time-dependent manner. It appears that the type of primary cells is of lesser importance, although the LDH test indicates that neurons are susceptible to lower concentrations of PS-NPs than astrocytes. Therefore, we further investigated the cytotoxic effect of PS-NPs in mixed cultures. After the first day of exposure, concentrations of 50 µg/mL and 25 µg/mL were found to significantly reduce cell viability in the MTT test, with the effect persisting to the next day. However, after 72 h of exposure, all tested concentrations of PS-NPs were found to be significantly toxic (Fig. 6C). Cell viability ranged from 93 to 84% over a concentration range of 0.5 to 50 µg/mL. In the case of the LDH test, a statistically significant early cytotoxic effect (24 h) of exposure was observed only at a concentration of 50 µg/mL (17% vs. control; *p* < 0.01). After prolonged (48 h) exposure, cell viability was found to be significantly reduced in the presence of 25 µg/mL PS-NPs. The greatest cytotoxicity occurred after 72 h, at concentrations of 50 µg/mL and 25 µg/mL, and was 23% (*p* < 0.0001) and 20% (*p* < 0.001), respectively (Fig. 6F).

This result can be explained by the higher cell density in co-cultures than in mono-cultures (Suppl. 1). In more dense cultures, cytotoxicity can be intensified by the release of chemicals that induce further toxicity in adjacent cells. An alternative explanation may be the loss of neuroprotective functions by astrocytes, accelerating neuronal damage, but this requires further detailed study.

### Delayed overactivation of astroglia under PS-NPs exposure

Considering the fact that astroglia play a supporting role for neurons and their dysfunction may be detrimental to brain health^29^, we investigated the temporal changes in astrocyte activation under conditions of PS-NPs exposure. We focused on glial fibrillary acidic protein (GFAP), which is a key component of astrocyte intermediate filaments and serves as the most commonly used marker to identify astrocytes and their activation state^30^.

**Fig. 7:**
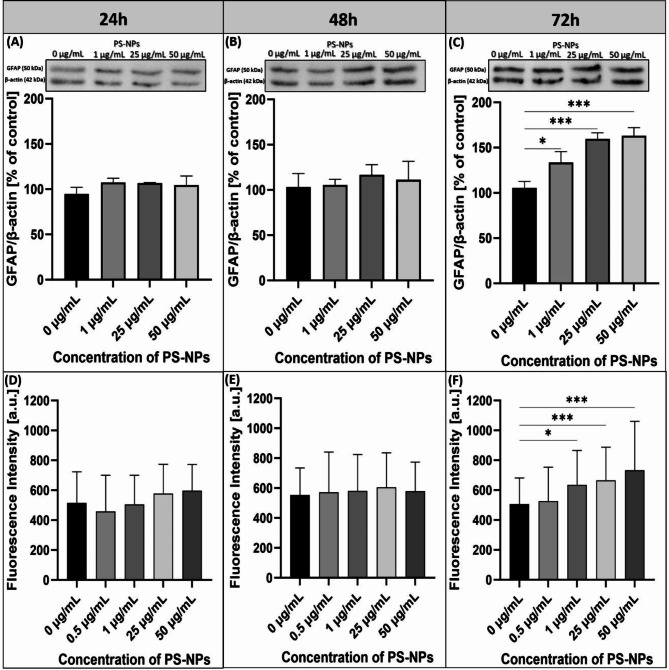
Time- and concentration-dependent overactivation of primary astrocytes exposed to PS-NPs. Graphs demonstrating the relative expression of GFAP normalized to β-actin (**A**–**C**) with representative immunoblots. The results are means ± SD of three independent experiments done using distinct cultures. The relative intensity of GFAP fluorescence (D–F) measured from confocal microscopy images after 24 h (**D**), 48 h (**E**) and 72 h (**F**) of exposure. The results are means ± SD of three independent experiments done using distinct cultures (total number of fluorescence intensity measurements in each groups = 15–20). The statistical analysis of the results was performed using the one-way ANOVA followed by Dunnett’s post hoc test (W–B) and Kruskal Wallis test followed by Dunn’s post hoc test (fluorescence intensity); *p < 0.05; ***p < 0.001.

**Fig. 8:**
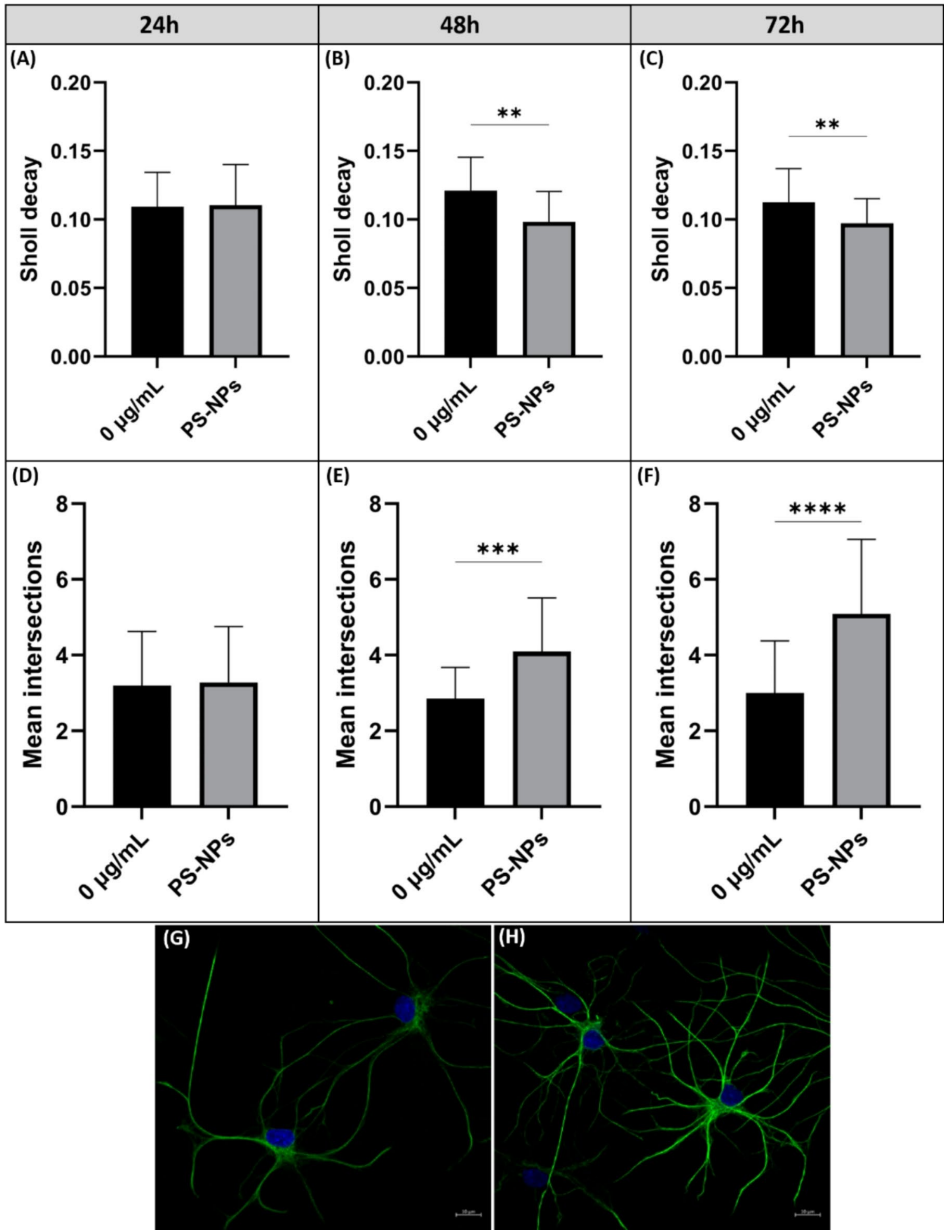
Sholl analysis quantifying morphological changes in control primary astrocytes and astrocytes exposed to 50 µg/mL PS-NPs for 24–72 h. The analysis was performed based on confocal microscopy images of astrocytes labelled with anti-GFAP antibody. Sholl decay (**A**–**C**) and mean intersections (**D**–**F**) were calculated using a web-based tool. Confocal microscopic images showing control (**G**) and PS-NP-treated (**H**) astrocytes with hypertrophic features visible after 72 h of exposure. The results are means ± SD of min. 20 cell images; **p < 0.01; ***p < 0.001; ****p < 0.0001 (Sholl decay (**A**–**C**) was analyzed using an unpaired t-test, mean intersections (**D**, **F**) with the Mann–Whitney test, and (**E**) with an unpaired t-test).

The results did not reveal significant changes in cells after 24 h and 48 h of exposure to PS-NPs (Fig. 7A, B). The relative GFAP concentrations assessed by W-B were not significantly different compared to the respective controls. We also did not observe enhanced GFAP immunoreactivity in confocal microscopy (Fig. 7D, E). However, a significant increase in the protein level of GFAP was noted after prolonged treatment of cells (for 72 h) with PS-NPs at concentrations of 1, 25 and 50 µg/mL (Fig. 7C) (*p* < 0.05; 0.001 vs. control). Furthermore, GFAP immunostaining in exposed primary astrocytes was found to be more intense after 72 h of exposure to PS-NPs (Fig. 7F) and the cells exhibited a hypertrophic appearance compared with the controls (Fig. 8), suggesting activation of the cells. Sholl analysis was performed to quantify the observed changes. Based on confocal microscopy images of astrocytes labeled with anti-GFAP antibody, parameters related to Sholl analysis, such as Sholl decay and mean intersections, were calculated using a web-based tool. The Sholl decay coefficient, which provides information on the rate at which the number of cell branches decreases with distance from the cell’s perikaryon (the lower the value, the greater the cell branching), decreased significantly at 48 h and 72 h after exposure (Fig. 8B–C). In turn, a significant increase in the mean intersections, the value defining the number of intersections of cell processes with the circles of increasing radii, was observed after 48 h and 72 h of exposure to PS-NPs (Fig. 8E–F). As shown in the confocal images, astrocytes exhibited an activated morphology, characterized by an increased number of branches (Fig. 8H). This morphological transformation is indicative of reactive astrogliosis associated with exposure to PS-NPs and the subsequent cellular response. The results of the Sholl analysis indicate that the activation of astrocytes occurs faster than indicated by GFAP fluorescence measurements and W-B analysis (48 h vs. 72 h).

## Discussion

The cytotoxicity of nanomaterials depends upon their physicochemical properties such as size, shape and functionalization of the surface, which is related to the surface charge characterized by the zeta potential. This factor is important for the stability of nanoparticles in suspension and for the initial adsorption of nanoparticles onto the cell membrane (for a review see:^31^. Our measurements of zeta potential revealed that the charge of both tested types of PS-NPs was negative at the applied temperatures, pH gradient and medium, indicating that the PS-NPs are stable and do not agglomerate. The highest zeta potential (− 25 mV) was observed for labelled nanoparticles at pH 8.3 and 37°C, while the lowest zeta potential (− 40 mV) for unlabelled PS-NPs was observed at pH 2.4 and 25°C. Under physiological conditions (37°C and pH 7.4), the zeta potential of fluorochrome-labelled PS-NPs was less negative than that of unlabelled PS-NPs.

Analysis of zeta potential of different NPs indicates interactions between biological systems and exogenous molecules^32–34^. In the case of PS-NPs, it has been shown using in vitro intestinal cell models that negatively charged 50 nm PS-NPs have more than 30-fold greater intracellular translocation potency than positively charged PS-NPs. In addition, size has a major impact on the ability to penetrate the cell, with 100 nm NPs showing a penetration rate of 0.8% and 50 nm NPs showing a penetration rate of 7.8%^35^. In a gastric adenocarcinoma cell line, smaller PS-NPs (44 nm) were found to accumulate faster in the cytoplasm than larger PS-NPs (100 nm)^36^.

During translocation, nanoparticles can be transported into and out of cells via mechanisms of endocytosis and exocytosis, with pinocytosis being the most prevalent form of endocytosis, involving the formation of vesicles containing the engulfed particles within the cytosol. Additionally, NPs can passively cross the cell membrane or be phagocytized^8^. Regarding aminated PS-NPs, most studies suggest that the primary route of their uptake is endocytosis. After internalization, early endosomes transport the NPs across the cell membrane to the endoplasmic reticulum^37–39^ and lysosomes^28,40^.

Since the adsorption of NPs onto the cell surface depends on the zeta potential, the rate of endocytotic uptake depends on the particle size. Thus, both factors determine the toxicity of nanoparticles^41^. Our results show that increased concentrations of PS-NPs and the time of exposure significantly enhance the efficiency of PS-NPs internalization in both astrocytes and neurons. However, in addition to the individual characteristics and physico-chemical properties of NPs, biological factors that vary across cell types may also influence nano-bio interactions. What we observed in our study, was a significantly higher rate of PS-NPs uptake in primary astrocytes compared to neurons, indicating a cell type-dependent internalization of PS-NPs (Fig. 2). The level of green fluorescence originating from labelled PS-NPs was found to be several-fold higher in primary astrocytes (Figs. 3, 4), suggesting a much higher intracellular concentration of PS-NPs. This observation is of great importance for further interpretation of the cytotoxic effect of PS-NPs and assessment of the sensitivity of various cell types to their toxic impact. Confocal images indicate a greater number of labelled dots representing clusters of internalized NPs in astrocytes (Fig. 2B, C) compared to neurons where they are smaller and sparse (Fig. 2E, F). The presence of large deposits in astrocytes suggests the involvement of phagocytosis in transport of NPs, as these cells have been reported to engulf even large parts of cells, such as neuronal synapses^42^. Indeed, we confirmed that the increased accumulation of PS-NPs is due to the phagocytic capacity of astroglia (Fig. 5), which may constitute an additional mechanism of internalization besides endocytosis. Astrocytes have been shown to exhibit their phagocytic activity both in health and disease (for a review see:^42^), although knowledge of the molecular mechanisms underlying this phenomenon in astrocytes remains limited compared to that for microglia. Therefore, the added value of our results is the demonstration of phagocytic activity of primary astrocytes under conditions of PS-NPs toxicity.

In contrast to our results, Murali et al.^43^ found that neurons did not internalize PS-NPs in the diameter range of 45–70 nm, which accumulate exclusively in microglia. This suggests that the mechanism of phagocytosis may predominate over endocytosis in the transfer of NPs into specific types of brain cells such as glial cells.

Analyzing our results from the perspective of the PS-NPs concentrations used, it is evident that cytotoxicity is significantly induced in a concentration range of 1–50 µg/mL in both astrocytes and neurons and the percentage of dead cells is similar. Moreover, longer exposure times provide a stronger effect. The conclusion might be that both cells are roughly equally sensitive to PS-NPs. However, the actual intracellular concentration of PS-NPs is several times higher in astrocytes than in neurons, indicating that astrocytes are more resistant, and the cytotoxic effect occurs when the internal presence of PS-NPs reaches a critical level.

During the past few years of mechanistic in vitro research on the cytotoxicity of nanoplastics and microplastics, the main approach has been to use of cell lines of various origin and, to a lesser extent, the primary cell cultures. Studies were performed using human astrocytoma 1321N1 cells, human liver cancer cells (HepG2), and human embryonic kidney HEK293 cells, to which PS-NPs (50 nm) were added at various concentrations (0.3–100 mg/mL) for 24 and 72h. These studies revealed damage to the cell membrane and cytotoxic effects^44^. Significant cell death was also noted in human neuroblastoma SH-SY5Y cells exposed to PS-NPs cells^4^ and in human gastric adenocarcinoma cells (AGS), where 44 nm PS-NPs were found to rapidly and efficiently accumulate in the cytosol, affecting the viability and morphology of these cells^36^. A significant decrease in the survival of human cervical cancer cells (HeLa) exposed to < 50 nm PS-NPs at concentrations of 1.25 mM and higher was also reported^14^. In contrast, research conducted by Schirinzi et al.^45^ on HeLa cells and cerebral glioblastoma T98G cells exposed to PS-NPs ranging from 40 to 250 nm in diameter at concentrations ranging from 10 ng/mL to 10 mg/mL indicated that cell viability is not affected^45^. Similarly, Jung et al.^46^ have found that the HEK293T cell line, as well as proliferative mouse cells (mouse embryonic fibroblasts and astrocytes), are not affected by PS-NPs exposure up to 200 mg/L as assessed by an MTT assay, although this exposure significantly reduces the viability of mixed neuronal cells isolated from mouse brain.

Our results were obtained using primary cultures, which are more representative of *in viv*o biology because they retain many of the characteristics and functions of the tissue from which they originate. These data complement the results of a few previous studies using primary cells of brain origin. Consequently, the collective data indicate that the type of cells exposed may be more important than expected in the context of cytotoxicity of nanoplastics, and that in nano-bio interactions, the significance of parameters of biological components may outweigh the significance of the characteristics of the nanomaterials. The observed adverse effects of PS-NPs are primarily a result of internalization efficiency, the number of particles taken up by a specific cell and their intracellular concentration, and then of nanomaterial-dependent parameters such as size.

Although there are discrepancies between the results of the studies on the relationship between the toxic effects of PS-NPs and their associated physico-chemical factors, there is consensus that PS-NP-induced cytotoxicity begins with the generation of reactive oxygen species (ROS) after endocytosis of NPs and the induction of oxidative stress that leads to the release of inflammatory factors and triggering of apoptosis^14,37,45,47^.

Here we demonstrate that PS-NPs added to cultured primary astrocytes induce a cytotoxic effect, reducing cell viability as assessed by MTT and LDH tests. The observed toxic effect is associated with a greater ability of astrocytes to accumulate PS-NPs compared to neurons. We therefore investigated whether this high internalization potency results in cellular reactivity, which is a common response of astrocytes to pathological insults. We examined the temporal pattern of changes in the expression of the astrocyte-specific protein GFAP, which is a marker of cellular activity indicating alterations in the cytoskeleton^48^. In the activated state, astrocytes undergo morphological, molecular, and functional alterations in response to pathological conditions^49^ and one of the characteristics is overexpression of GFAP. In our study, overexpression of this protein was confirmed by both qualitative (immunocytochemistry) and semi-quantitative analyses (W-B). In addition to the more intense GFAP immunolabelling, PS-NP-exposed astrocytes are morphologically different and have a hypertrophic appearance. Sholl analysis confirmed the increased number of cellular processes after exposure to PS-NPs (Fig. 8). Using this tool we can assess the level of astrocytes activity^50^.

Interestingly, the response of astrocytes to toxic conditions was delayed in time and overexpression of GFAP was observed only after prolonged exposure to PS-NPs-treated astrocytes, i.e. after 72 h, whereas Sholl analysis indicates that increased branching of PS-NP-exposed astrocytes starts already after 48 h of exposure. These results coincide well with the observed high rate of PS-NPs accumulation, suggesting that astrocytes act as PS-NPs depots and become activated when the storage of NPs reaches a critical level. The results are partially consistent with those by Jung et al.^46^ who found that PS-NPs (100 nm) at concentrations up to 200 mg/L induce overexpression of GFAP, but do not affect the viability of astrocytes.

Astrocytes play a variety of roles to support neurons and maintain homeostasis in the central nervous system (CNS). The proper functioning of these cells in their relationships with neurons and other cells is fundamental to the physiology of the brain. The loss of protective functions or the acquisition of abnormal activity of astrocytes determines the degree of neuronal impairment and death and has been connected with a variety of brain disorders such as neurodegenerative diseases, multiple sclerosis, and ischemia^29,51,52^. In addition, environmental neurotoxicants such as lead (Pb) have been shown to preferentially accumulate in astroglia^53^, impairing cell functions and glial-neuronal interactions^54^. Similarly, extensive accumulation of PS-NPs in astrocytes may result in cellular dysfunction and reduced neuroprotective and/or acquisition of neurotoxic properties. However, this aspect requires further study.

In conclusion, the results of the current studies show that PS-NPs (25 nm) are internalized into primary astrocytes and neurons in a manner, which depends on the time of exposure and the concentration of PS-NPs. This results in reduced cell viability and increased release of LDH in both cell types. We show for the first time that PS-NPs are significantly retained in astrocytes over time and the scale of this retention is several times greater than that observed in neurons. This significant accumulation of PS-NPs is due to the mechanism of phagocytosis. We also provide morphological and biochemical evidence that the prolonged (72 h) cell-dependent accumulation rate strongly influences the cytotoxic effect of PS-NPs and simultaneously promotes activation of astrocytes, as indicated by overexpression of the GFAP marker and morphological analysis of cell branching. The collective mechanistic data from our in vitro studies may suggest that in the brain, astroglia can accumulate and store large amounts of PS-NPs, thus demonstrating their neuroprotective function.

## Electronic supplementary material

Below is the link to the electronic supplementary material.

Supplementary Material 1

Supplementary Material 2

## Data Availability

The datasets generated and analysed during the current study are available at the RepOD repository 10.18150/P2WGQ0.
