# Supplementary material for: Primary astrocytes as a cellular depot of polystyrene nanoparticles
Source: Sci Rep. 2025 Feb 22;15:6502. doi: 10.1038/s41598-025-91248-w (PMC11846901; doi:10.1038/s41598-025-91248-w)

**Westernblot – Astrocytes (GFAP)**

The image shows the original Western blot. The upper membrane contains lysate samples from astrocytes in the following order: Control, 1, 25, and 50 µg/mL (after 24 h exposure), followed by Control, 1, 25, and 50 µg/mL (after 48 h exposure). The lower membrane presents duplicate samples after 72 h exposure, in the sequence: Control, 1, 25, 50 µg/mL, and again Control, 1, 25, 50 µg/mL.

The upper bands correspond to GFAP, while the lower bands correspond to β-actin.


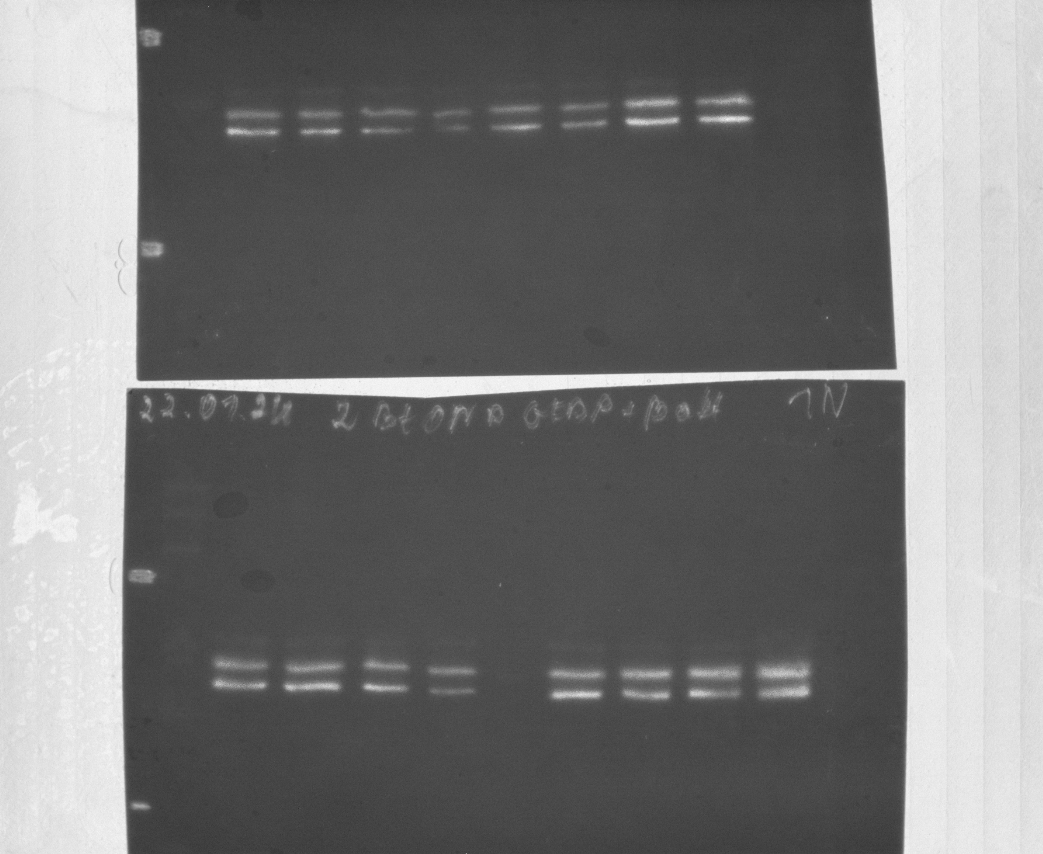

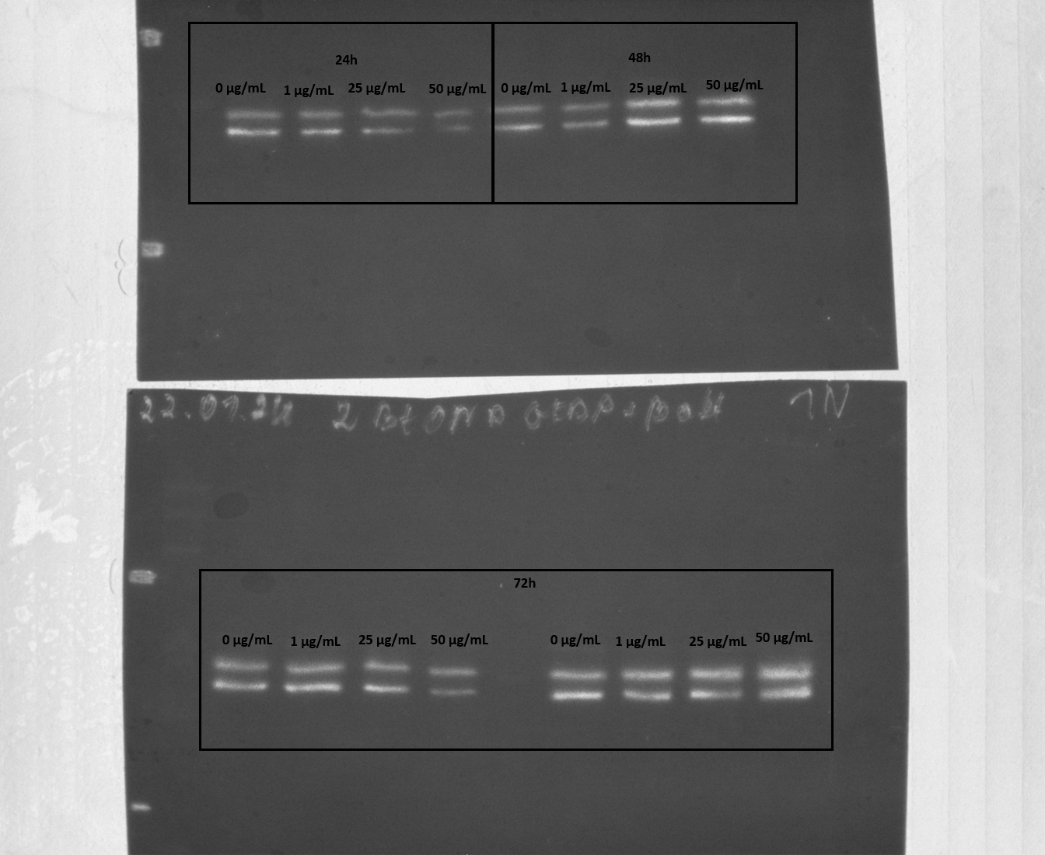

Supplement: Supplementary file 1 — Supplementary Material 1 [file 41598_2025_91248_MOESM1_ESM.docx]
