# Supplementary figures and images for: Primary astrocytes as a cellular depot of polystyrene nanoparticles
Source: Sci Rep. 2025 Feb 22;15:6502. doi: 10.1038/s41598-025-91248-w (PMC11846901; doi:10.1038/s41598-025-91248-w)

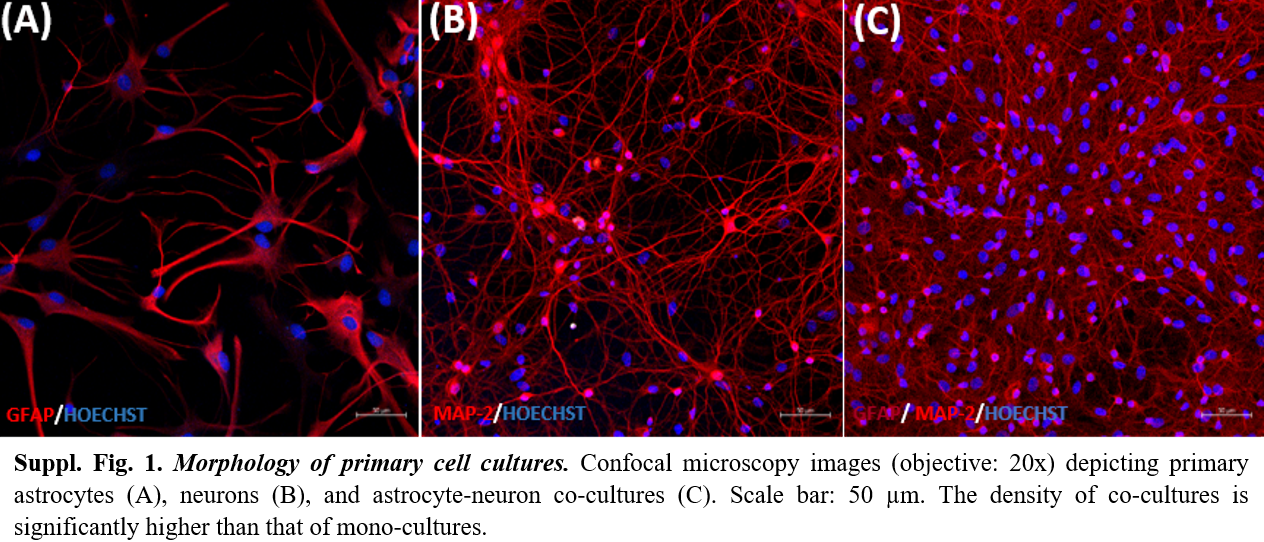

Supplement: Supplementary file 2 — Supplementary Material 2 [file 41598_2025_91248_MOESM2_ESM.tif]
